# Kinesiophobia Levels in Patients with Parkinson’s Disease: A Case-Control Investigation

**DOI:** 10.3390/ijerph18094791

**Published:** 2021-04-30

**Authors:** Ana María Jiménez-Cebrián, Ricardo Becerro-de-Bengoa-Vallejo, Marta Elena Losa-Iglesias, Carmen de Labra, César Calvo-Lobo, Patricia Palomo-López, Eva María Martínez-Jiménez, Emmanuel Navarro-Flores

**Affiliations:** 1 Department Nursing and Podiatry, Faculty of Health Sciences, University of Malaga, Arquitecto Francisco Peñalosa 3, Ampliación de Campus de Teatinos, 29071 Málaga, Spain; amjimenezc@uma.es; 2 Instituto de Investigación Biomédica de Málaga (IBIMA), 29010 Málaga, Spain; 3 Facultad de Enfermería, Fisioterapia y Podología, Universidad Complutense de Madrid, 28040 Madrid, Spain; ribebeva@ucm.es (R.B.-d.-B.-V.); cescalvo@ucm.es (C.C.-L.); eva.hache2@hotmail.com (E.M.M.-J.); 4 Faculty of Health Sciences, Universidad Rey Juan Carlos, 28922 Alcorcon, Spain; marta.losa@urjc.es; 5 NEUROcom, Faculty of Nursing and Podiatry, University of A Coruna, 15006 A Coruña, Spain; 6 Facultad de Podología, University Center of Plasencia, Universidad de Extremadura, 10600 Plasencia, Spain; patibiom@unex.es; 7 Department of Nursing, Faculty of Nursing and Podiatry, Frailty Research Organized Group, Universidad de Valencia, 46010 Valencia, Spain; emmanuel.navarro@uv.es

**Keywords:** movement disorders, Parkinson’s disease, musculoskeletal and neural physiological phenomena

## Abstract

Background: Kinesiophobia can be an obstacle to physical and motor activity in patients with Parkinson’s disease (PD). PD affects patients’ independence in carrying out daily activities. It also impacts a patient’s biopsychosocial well-being. The objective of this study was to analyze the levels and scores of kinesiophobia in PD patients and compare them with healthy volunteers. Methods: We deployed a case-control study and recruited 124 subjects (mean age 69.18 ± 9.12). PD patients were recruited from a center of excellence for Parkinson’s disease (cases n = 62). Control subjects were recruited from the same hospital (control n = 62). Kinesiophobia total scores and categories were self-reported using the Spanish version of the Tampa Scale of Kinesiophobia (TSK-11). Results: Differences between cases and control groups were analyzed using the Mann–Whitney U test. Statistically significant differences (*p* < 0.05) were shown between groups when comparing kinesiophobia categories (or levels) and total scores, revealing higher kinesiophobia symptoms and levels in PD patients. All of the PD patients reported some degree of kinesiophobia (TSK-11 ≥ 18), while the majority of PD patients (77.3%) had kinesiophobia scores rated as moderate to severe (TSK-11 ≥ 25). On the other hand, ~45.1% of controls reported no or slight kinesiophobia and 53.2% reported moderate kinesiophobia. Conclusions: Total kinesiophobia scores were significantly higher in PD patients compared with healthy controls, with moderate to severe kinesiophobia levels prevailing in PD patients. Therefore, individuals living with PD should be evaluated and controlled in order to detect initial kinesiophobia symptoms.

## 1. Introduction

Kinesiophobia is a psychological factor that manifests as an excessive fear of movement or physical activity. Kinesiophobia patients believe that movement can cause re-injury and additional pain [1]. A characteristic behavior of kinesiophobia is avoiding actions that cause fear, with motor passivity as a symptom of this problem [2,3]. In the long-term, kinesiophobia is associated with a decrease in physical condition, avoidance of physical activity, functional disability, and depression [4,5]. Kinesiophobia prevalence data varies according to what population group is being assessed. For example, it is found in 62% of patients after the reconstruction of the anterior cruciate ligament in the knee, 66.6% of patients with lupus erythematosus, and 75.1% of patients with fibromyalgia [6,7,8].

Various authors have reported a relationship between kinesiophobia and general psychological problems as a common feature in subjects with hallux valgus deformities [9], cardiovascular pathology [3], fibromyalgia syndrome [8], lupus erythematosus [7], knee replacement [10], a reconstructed anterior cruciate ligament [6], chronic fatigue syndrome [11], as well as chronic back and musculoskeletal pain [12,13,14].

Likewise, we intuited that this disorder would be present in PD patients. The literature shows that kinesiophobia is an attribute that creates limitations regarding quality of life and total energy expenditure [15]. This debilitating fear of movement can also be considered a factor that intensifies one’s limited activity levels. At all times, it is important to bear in mind that moderate physical activity has a positive impact on PD and should be used as a treatment to alleviate symptoms such as motor dysfunction, cognitive deficits, and depression [16].

For PD patients, kinesiophobia can be an obstacle to physical and motor activity, as well as to independence in carrying out daily activities that impact biopsychosocial well-being. Accordingly, it is necessary to diagnose this disorder in PD patients so that therapeutic attention can noy only focus on reducing the fear of activity but also maintaining independence and avoiding a sedentary lifestyle. Since there are no studies focusing on this psychological factor in PD patients when compared with a control group, our study’s objective was to analyze the levels of kinesiophobia in PD patients and compare them with volunteers without PD. We hypothesized that kinesiophobia scores between people with PD could be matched with neurologically healthy controls.

## 2. Material and Methods

### 2.1. Design and Sample

A descriptive and observational case-control study was carried out in a center of excellence for Parkinson’s patients in Malaga (Spain) between October and December 2020. A sample of 124 subjects completed the study and they were divided into persons with Parkinson’s disease (for case group, n = 62) and a matched-paired of non-PD subjects (for the control group, n = 62). They were recruited by a consecutive sampling method using a successive and non-aleatorized simple method. Subjects with PD were enlisted from the center of excellence for Parkinson’s disease and control subjects were recruited from the same hospital. Participation selection and inclusion criteria were as follows: (1) age 50 or higher, due to the fact that this group is likely to more frequently suffer from falls than younger PD and healthy subjects; (2) walking autonomously with or without a walking aid; (3) persons with PD (case group) diagnosed according to specialist of neurology criteria and persons without PD (control group), the diagnosis, and signs suggestive of the disease. The rules for exclusion of subjects were a (1) refusal to supply informed consent and an (2) inability to understand and carry out the study instructions.

### 2.2. Procedure

Baseline measurements included general questions associated to (1) demographic variables (e.g., age, weight, height, time since PD diagnosis) and (2) characteristics about comorbid conditions (e.g., diabetes, obesity, musculoskeletal difficulties, vascular disorders).

Next, participants completed the Spanish Tampa Scale for Kinesiophobia (TSK-11) [17]. The scale is a psychometric tool for diagnosis, prognosis, follow-up, and clinical orientation [18,19] and it is used to assess a patient’s fear of reinjury due to movement. It consists of 11 items, each scored 1–4. Patients indicate their degree of agreement with each of the presented statements using a Likert-type scale from 1 (totally disagree) to 4 (totally agree). This questionnaire is also designed to assess the avoidance dimensions of activity and harm. Yet, in this work, we only used the total score, which has a minimum value of 11 points and a maximum value of 44 points. High scores indicate greater fear of movement, i.e., higher kinesiophobia. In addition, TSK-11 total scores were categorized into kinesiophobia levels of fear of movement, including no fear of movement (11–17 points), slight fear of movement (18–24 points), moderate fear of movement (25–31 points), severe fear of movement (32–38 points), and maximum fear of movement (39–44 points) [9].

Adequate psychometric properties were reported for this scale, showing an internal consistency of 0.78 with Cronbach’s α and a test–retest with ICC of 0.82. The validity and reliability of the scale in the Spanish version was completed by Gomez-Perez et al. [20].

### 2.3. Ethical Considerations

The current study was approved by the Bioethics and Biosafety Committee (approval number 1450610) at the University of Valencia (Spain, 2020). All volunteers gave written informed consent before taking part in this investigation. Human and ethical experimentation standards of the Declaration of Helsinki (World Medical Association) and other organizations were respected at all times.

### 2.4. Sample Size Calculation

The sample size was calculated with specific levels of confidence, power, and groups of equal size using the Epidat 4.2 Program (Consellería de Sanidade, Xunta de Galicia, Spain; Organización Panamericana de la salud (OPS-OMS), Universidad CES, Colombia). A total sample size of 122 participants (61 per group) was established with a confidence level of 70%, a power of 0.80, an odds ratio of 2.0, and an expected exposure proportion of 66.67% in the PD group, as well as 50% in the controls. The total sample (124 participants) consisted of 62 cases (38 men and 24 women) and 62 controls (37 men and 25 women).

### 2.5. Statistical Analysis

Statistical analysis was realized through the 25.0v tware (IBM Corp., Armonk, NY, USA) with an alpha error of 0.05 for a 95% confidence interval (CI).

Regarding quantitative data, the Kolmogorov–Smirnov test was used to evaluate normality. All data were dispensed as parametric tests (the Student’s *t*-test showed a *p*-value lower than 0.05) and were described as the median ± standard deviation (SD) and range (minimum–maximum). This contrasted between groups, as it was compared by bot the Student’s *t*-test and the Mann–Whitney U tests using independent samples.

Concerning categorical data, frequencies and percentages were applied to distinguish these values. Differences between groups were contrasted using the Chi-squared test (BDI category).

## 3. Results

### 3.1. Descriptive Data

A sample of 124 subjects completed the study. They were divided into persons with PD (for case group, n = 62) and matched-paired participants without PD (for the control group, n = 62). Ages ranged between 50–84 years old. Cases and controls were matched for age, sex, and BMI. Statistically significant differences were not shown (*p* > 0.05) between groups for the descriptive data (Table 1).

### 3.2. Outcome Measurements

Table 1 shows that, generally speaking, 100% (n = 124) of participants reported the expected characteristics. Furthermore, 54.8% of the patients presented predisposing factors, including 21.8% (n = 27) vascular disease, 21.8% (n = 27) osteoarticular pathology, 9.7% (n = 12) diabetes, and 4% (n = 5) obesity.

Table 2 shows that, all of the PD patients reported some degree of kinesiophobia (TSK-11 ≥ 18), and the majority of PD patients (77.3%) had kinesiophobia scores rated as moderate to severe (TSK-11 ≥ 25). On the other hand, ~45.1% of controls reported no or slight kinesiophobia and 53.2% reported moderate kinesiophobia.

## 4. Discussion

In our study, the objective was to analyze the levels of kinesiophobia in PD patients and compare them to volunteers without PD. Our hypothesis was that kinesiophobia scores between people with PD would align with neurologically healthy controls.

Hence, this study compared kinesiophobia scores obtained from 62 subjects with PD and 62 without PD (considered healthy control participants). The results found that the majority of the subjects with PD suffered from kinesiophobia on one of its four levels (slight, moderate, severe, or maximum). The data indicate that 43.5% suffered from a “moderate” level of fear of movement. Of PD patients, 77.3% presented high levels of kinesiophobia (Spanish version TSK-11 scores > 25).

With the obtained data, we could suggest that these results support our hypothesis that kinesiophobia scores between people with PD could be matched with neurologically healthy controls.

As a novelty, it should be noted that our study provides a comparison with a control group of non-Parkinson subjects, of whom 45.1% reported mild or no kinesiophobia and 53.2% reported moderate kinesiophobia.

In a study that carried out this research in patients with lupus erythematosus, 66.6% of patients presented high levels of kinesiophobia. This highlighted the condition’s relationship with high levels of depression [7]. In various other studies, similar results were obtained. In patients with fibromyalgia, 75.1% reported “maximum” kinesiophobia [8]. Moreover, 55.8% of patients undergoing knee replacement surgery presented high values of kinesiophobia [10]. Further, patients with a high degree of hallux valgus (HV) deformity showed increased levels of kinesiophobia. Statistically significant differences (*p* < 0.001) were shown for comparison of total kinesiophobia scores among varying degrees of HV deformity [9].

Therefore, this alteration of values in participants of our study was significantly higher in PD patients compared to the 194 healthy control participants (29.21 ± 5.49 vs. 23.90 ± 5.21). Similar to these results were the findings of Koçyïgït and Akaltun, who studied patients with fibromyalgia syndrome. Although fibromialgia patients share different pathophysiological mechanisms with PD patients, significantly higher kinesiophobila levels were found in fibromialgia patients and not in the healthy control group [8].

On the other hand, we found only one previous study of this disorder in PD patients, which was carried out by Sütçü et al., who evaluated this phenomenon in a group of 20 PD patients in association with fatigue and its relationship with quality of life, physical activity, and functional capacity. They reported that fatigue and high levels of kinesiophobia can negatively affect the independent performance of daily activities in PD patients [15]. However, their scores (39.85 ± 7.44) were evaluated with the Turkish version TSK-17 (whose values range from 17–68) as opposed to the shorter Spanish (TSK-11) used in our study (whose values range between 11 and 44). This prevents us from comparing our scores and theirs.

An important point to keep in mind is that the presence of this disease always has a negative influence on patients. A review of kinesiophobia measured using TSK in patients with neck pain highlighted that higher TSK scores predicted a longer duration of symptoms [21]. Similarly, Luque-Suarez et al., in a systematic review of kinesiophobia in patients with chronic musculoskeletal pain, showed that a higher degree of this problem was associated with higher levels of pain intensity and lower quality of life [22].

Likewise, in our PD patients, we observed that motor impairment caused alterations in gait and consequent risk of falls, leading to kinesiophobia that results from fear of falling [23]. PD therapists and researchers set a goal of avoiding and controlling gait disturbances to manage the risk of falls [24]. It is necessary to initially detect symptoms of kinesiophobia in PD patients to prevent them from physical inactivity [25,26] and to contribute to preventing them from falling [27]. Therefore, detection and monitoring of this disorder in PD patients should be used as a tool in the prevention of falls. In fact, there is now a new version of the Tampa Scale of Kinesiophobia that has been developed specifically for PD [28].

More studies are needed to analyze whether gait and balance alterations in PD patients favor the onset of kinesiophobia. Further research could determine whether the severity of these symptoms predicts kinesiophobia. It could also investigate the interventions that could improve said phobia.

Finally, we believe our study is important because it is the first to evaluate and classify kinesiophobia in PD patients while also including a control group. The research, however, does have some limitations. First, the sample size was small and thus it would have been beneficial to have included more participants to strengthen the results of the study, as well as to exclude participants who presented predisposing factors. Second, despite using a sample size calculation, it would have been useful to have carried out a prior pilot study to obtain better quality research. The breadth of the selection criteria is another limitation, as it favors the heterogeneity of the sample. To avoid this in future research, larger sample sizes and longitudinal evaluation of outcomes would help determine more definitive conclusions.

## 5. Conclusions

PD was found to have a negative impact on people’s kinesiophobia scores. Total kinesiophobia scores were significantly higher in PD patients compared with healthy controls. Moderate to severe kinesiophobia levels prevailed in PD patients. Therefore, PD individuals should be evaluated and controlled to detect initial kinesiophobia symptoms.

## Figures and Tables

**Table 1:** Descriptive data of the Parkinson patients and healthy matched-paired controls.

Descriptive Data		Total Group Mean ± SD Range (n = 124)	Cases Mean ± SD Range (n = 62)	Controls Mean ± SD Range (n = 62)	*p*-Value
Age (years)		69.18 ± 9.12 (50–84)	69.23 ± 9.15 (50–84)	69.13 ± 9.15 (50–84)	0.097 †
Weight (kg)		74.10 ± 14.84 (43–135)	73.36 ± 17.63 (43–135)	74.83 ± 11.49 (54–100)	0.582 †
Height (m)		1.67 ± 0.09 (1.47–1.91)	1.66.37 ± 9.64 (1.47–1.91)	1.67 ± 7.80 (1.47–1.85)	0.690 †
BMI (kg/m^2^)		26.61 ± 4.61 (16.16–40.31)	26.37 ± 5.24 (16.16–40.31)	26.85 ± 3.90 (19.83–35.43)	0.0563 †
Time since PD diagnosis (years)		N/A	11.83 ± 6.71 (0–32)	N/A	<0.001 †
Sex (%)	Male Female	75 (60.5%) 49 (39.5)	38 (61.3%) 24 (38.7%)	37 (59.7%) 25 (40.3)	0.854 ‡

Abbreviations: BMI, body mass index; N/A, not applicable; SD, standard deviation. In all analyses, *p* < 0.05 (with a 95% confidence interval) was considered statistically significant. Median ± interquartile range, range (min–max) and † Student’s *t*-test for independent samples were applied. ‡ Chi-squared test were used.

**Table 2:** Comparisons of TSK-11 scores and categories between patients with Parkinson’s disease (PD) patients and patients without PD (matched-paired control)s.

Outcome Measurements			Total Group (n = 124)	Cases Mean ± SD (n = 62)	Controls (n = 62)	*p*-Value (Cases vs. Controls)
TSK-11 Category *	Fear of movement	No	9 (7.3%)	0 (0%)	9 (14.5%)	<0.001 *
		Slight	33 (26.6%)	14 (22.6%)	19 (30.6 %)	
		Moderate	60 (48.4%)	27 (43.5%)	33 (53.2%)	
		Severe	19 (15.3%)	18 (29 %)	1 (1.6 %)	
		Maximum	3 (2.4%)	3 (4.8%)	0 (0 %)	
TSK scores			26.56 ± 5.96 (11–41)	29.21 ± 5.49 (18–41)	23.90 ± 5.21 (11–35)	**<0.001 †**

* TSK-11, Tampa Scale Kinesiophobia. Frequency, percentage (%) and Chi-squared test (χ^2^) were utilized. TSK-11 category were divided as follows: (1) 11 to 17 points: no fear of movement, (2) 18 to 24 points: slight fear of movement, (3) 25 to 31 points: moderate fear of movement, (4) 32 to 38 points: severe fear of movement, (5) 39 to 44 points: máximum fear of movement. †, TSK-11 scores, Median ± interquartile range, range (min–max) and Mann–Whitney U test were used. In all the analyses, *p* < 0.05 (with a 95% confidence interval) was considered statistically significant (bold).

## Data Availability

The dataset supporting the conclusions of this article is available in the Emmanuel.Navarro@uv.es in the Faculty of Nursing and Podiatry, Department of Nursing. University of Valencia. Frailty Research Organizaded Group. (FROG), Spain.
